# Supplementary material for: Protein quantitative trait locus study in obesity during weight-loss identifies a leptin regulator
Source: Nat Commun. 2017 Dec 12;8:2084. doi: 10.1038/s41467-017-02182-z (PMC5727191; doi:10.1038/s41467-017-02182-z)
Supplement: Supplementary file 2 — Supplementary Information [file 41467_2017_2182_MOESM2_ESM.pdf]

## Supplementary Figures

**a**

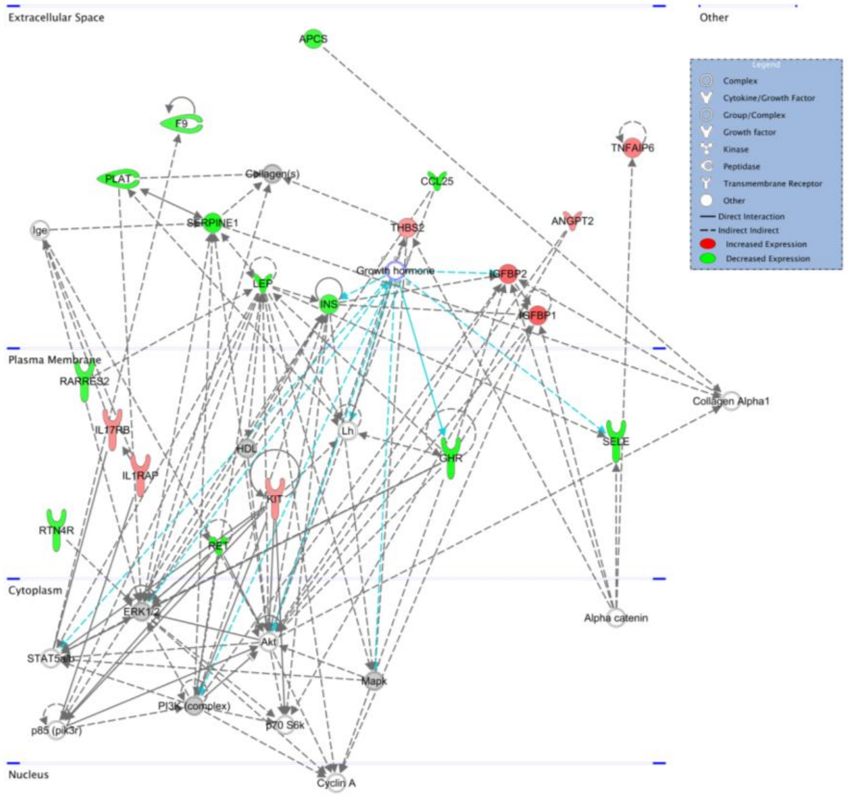


b

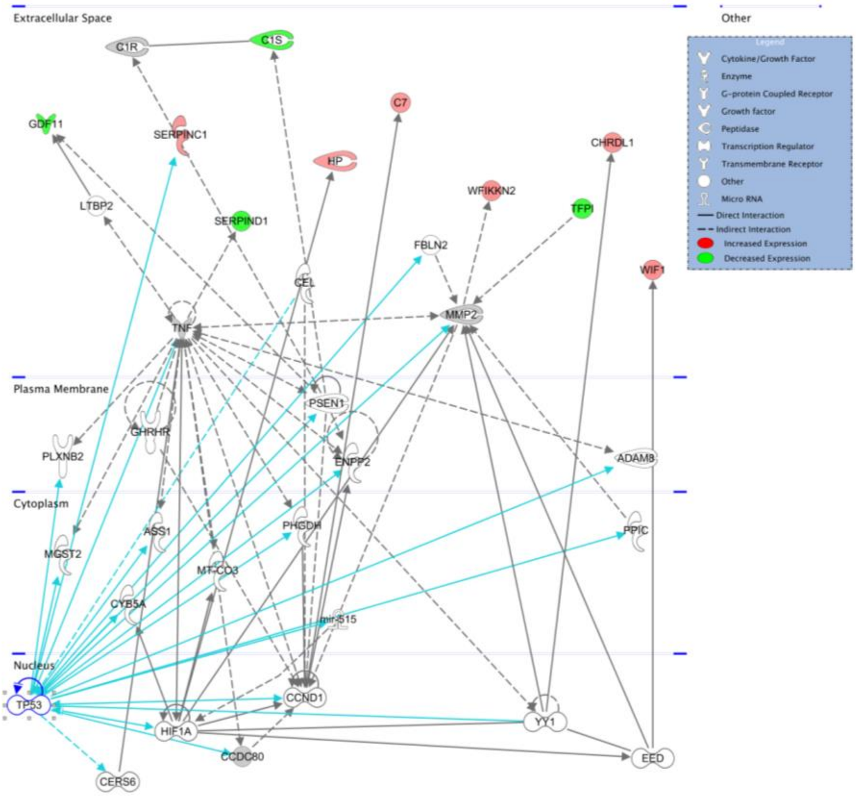

**c**

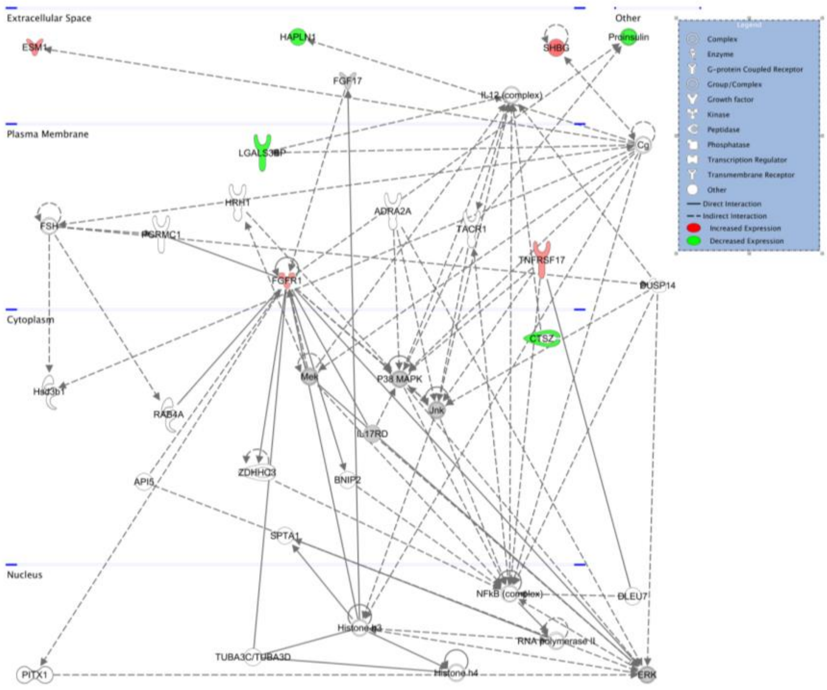


**Supplementary Fig. 1. Protein-protein interaction networks.** The network displays interactions between proteins related genes that were associated with BMI level at baseline and during LCD with indications on cellular and sub-cellular location. Solid and dashed lines indicate direct and indirect interactions, respectively. Using proteins expression fold change during LCD, genes coding for proteins with increased expression level are colored in shades of red while proteins with expression decreasing are colored in shades of green. Genes in grey were available in the list of 1,129 proteins but not in the subset of 42 proteins. White nodes were not available in our experiment and integrated into the networks using Ingenuity's Pathways Knowledge Base. In the current study, 3 major networks of interacting proteins were identified: (a) Network 1: connected genes were involved in tissue morphology, inflammatory response and cardiovascular diseases; (b) Network 2: connected genes were involved in organismal injury and abnormalities, embryonic development and organismal development; (c) Network 3: genes were involved developmental disorder, endocrine system disorders, hereditary disorder.

**a**

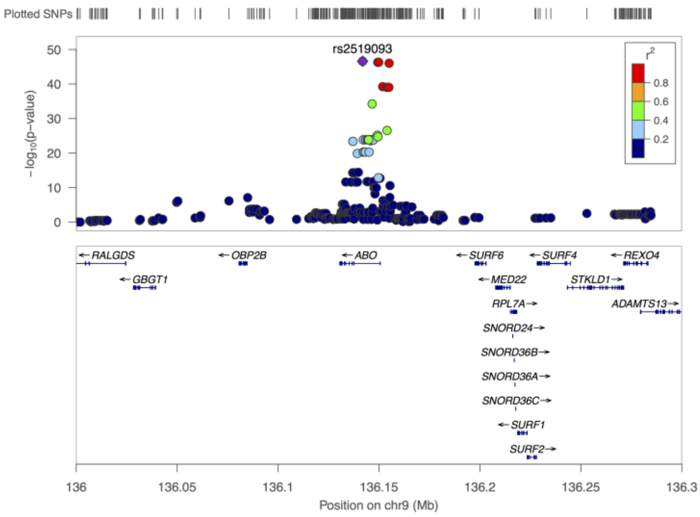


**b**

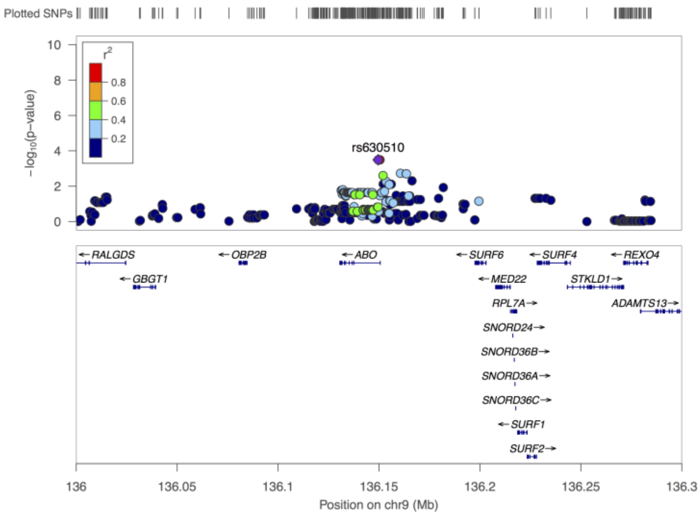


**Supplementary Fig. 2. Locus-specific plots for sE-Selectin *trans*-acting SNPs located within *ABO* gene.** Association plot at baseline for (a) *trans*-acting pQTL SNPs associated with sE-Selectin protein expression; (b) proximal SNPs associated with ABO gene expression. Genotyped and imputed SNPs were used to plot association signals in the region using LocusZoom. SNPs are plotted by their positions on chromosomes against $-\log_{10}$ *p*-value. Color for LD ($r^2$) with the lead SNP (purple diamond) is based 1000 genome CEU population data (hg19/1000 Genomes Mar 2012 EUR).

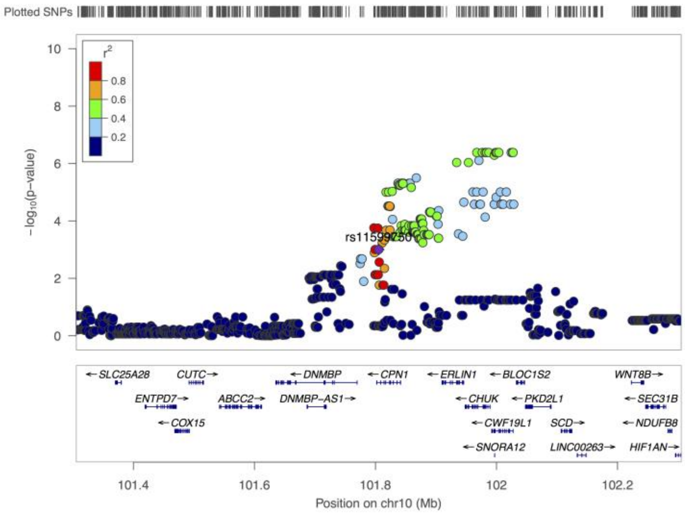


**Supplementary Fig. 3. Locus specific plot for CWF19L1 associated cis-eQTL SNPs at baseline.** Best trans-acting pQTL SNP (rs11599750) for calpastatin near association signal for CWF19L1 gene expression.

a

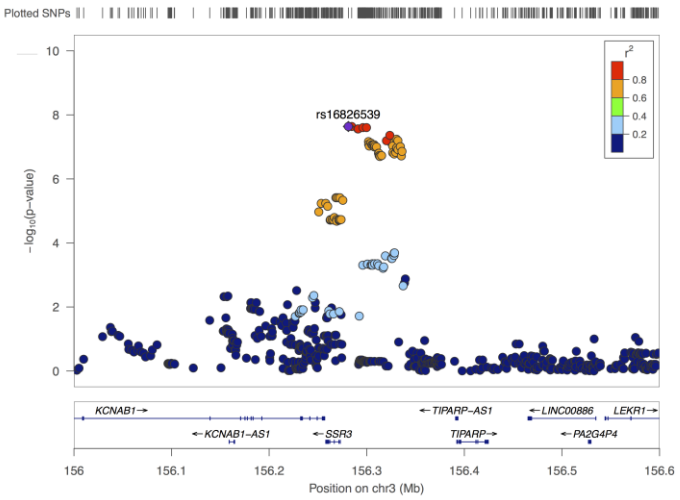

Plotted SNPs
rs16826539
r2
0.8
0.6
0.4
0.2
−log10(p−value)
KCNAB1→
←KCNAB1−AS1
←SSR3
←TIPARP−AS1
TIPARP→
←LINC00886
←PA2G4P4
LEKR1→
156
156.1
156.2
156.3
156.4
156.5
156.6
Position on chr3 (Mb)


b

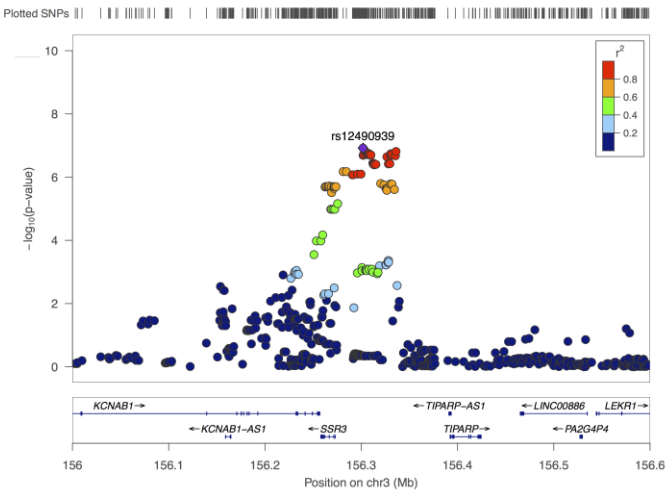

Plotted SNPs
rs12490939
r2
0.8
0.6
0.4
0.2
−log10(p−value)
KCNAB1→
←KCNAB1−AS1
←SSR3
←TIPARP−AS1
TIPARP→
←LINC00886
←PA2G4P4
LEKR1→
156
156.1
156.2
156.3
156.4
156.5
156.6
Position on chr3 (Mb)


c

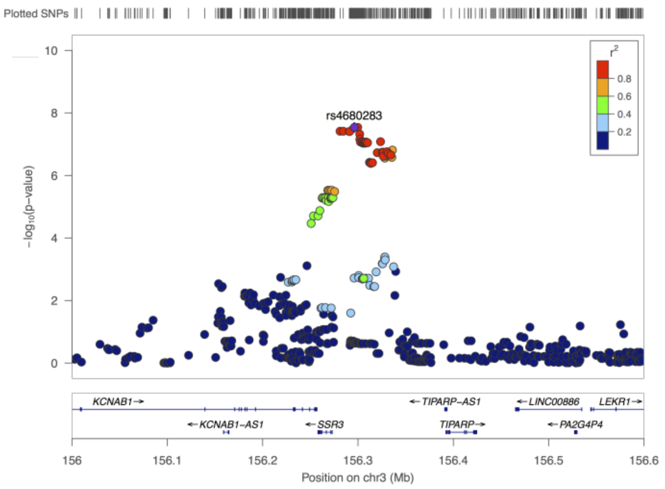

Plotted SNPs
rs4680283
r2
0.8
0.6
0.4
0.2
−log10(p−value)
KCNAB1→
←KCNAB1−AS1
←SSR3
←TIPARP−AS1
TIPARP→
←LINC00886
←PA2G4P4
LEKR1→
156
156.1
156.2
156.3
156.4
156.5
156.6
Position on chr3 (Mb)

d

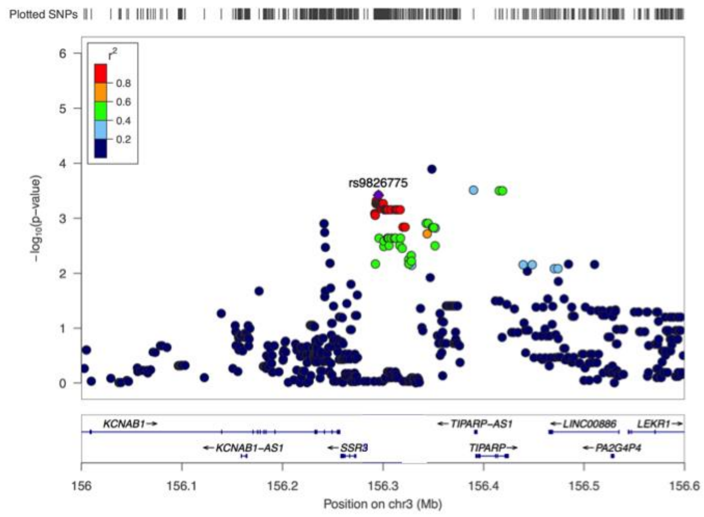


**Supplementary Fig. 4. Regional association plots generated within a region of *trans*-pQTL for PUR8, ferritin and CA1**. Association plot at baseline for (a) *trans*-pQTL SNPs associated with PUR8 protein expression, (b) *trans*-pQTL SNPs associated with ferritin protein expression, (c) *trans*-pQTL SNPs associated with CA1 protein expression and, (d) *cis*-eQTL SNPs associated with *TIPARP* gene expression. The region containing pQTL signal for PUR8, ferritin and CA1 between 156.28 and 156.34 Mb on chromosome 3 is highlighted in purple. In this region, SNP rs9826775 displayed the strongest association with *TIPARP* gene expression with p = 3.8 x 10-4.

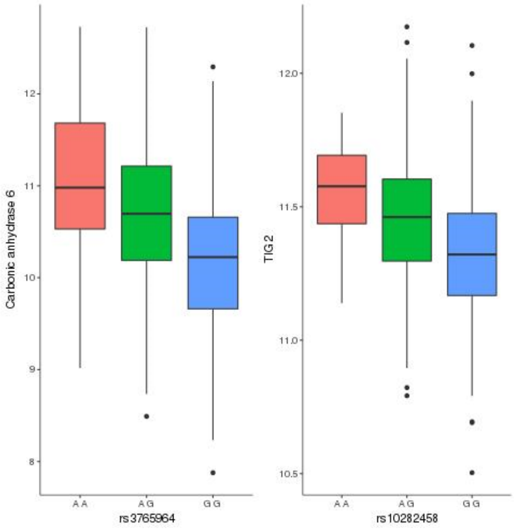


**Supplementary Fig. 5. Boxplots of baseline protein levels by genotype for best pQTL SNPs.** Boxplots for the genotypes of the two SNPs that are most significantly associated (rs3765964 and rs10282458) with carbonic anhydrase 6 (gustin) and TIG2 (chemerin)protein levels at baseline.

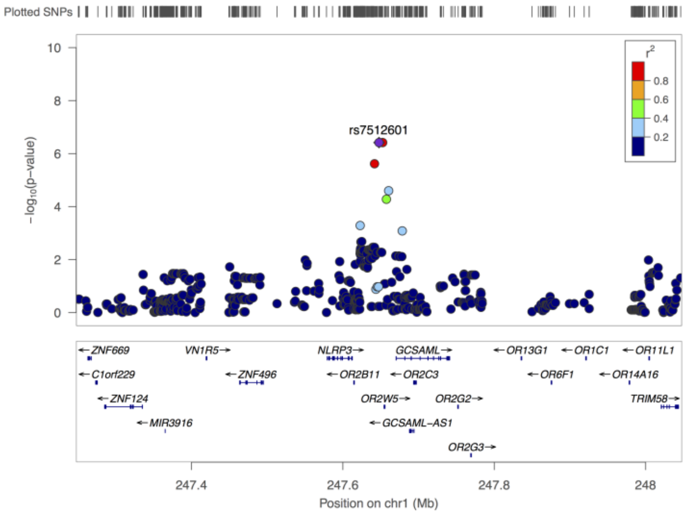


**Supplementary Fig. 6. Locus-specific plots for gustin associated *trans*-acting SNPs during LCD intervention.** Strongest association to protein level change during LCD estimated for SNP rs7512601 ($p = 3.8 \times 10^{-7}$). This SNP is located in the promoter region of olfactory receptor gene *OR2W5.*

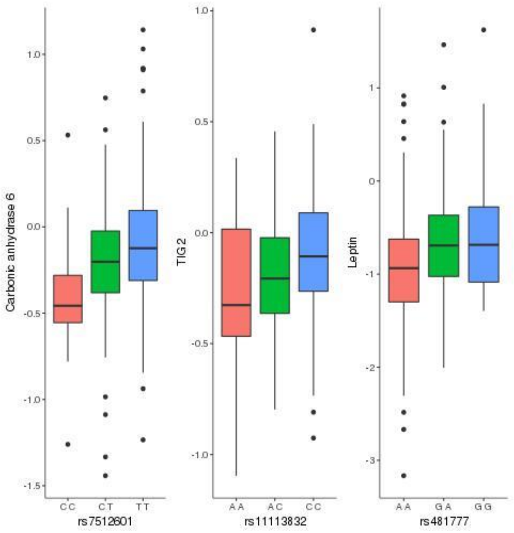


**Supplementary Fig. 7. Boxplots of LCD protein levels by genotype for best pQTL SNPs.** Boxplots of the genotypes of the three SNPs that are most significantly associated (rs7512601, rs11113832 and rs481777), respectively, with carbonic anhydrase 6 (gustin), TIG2 (chemerin) and leptin protein levels change during LCD.

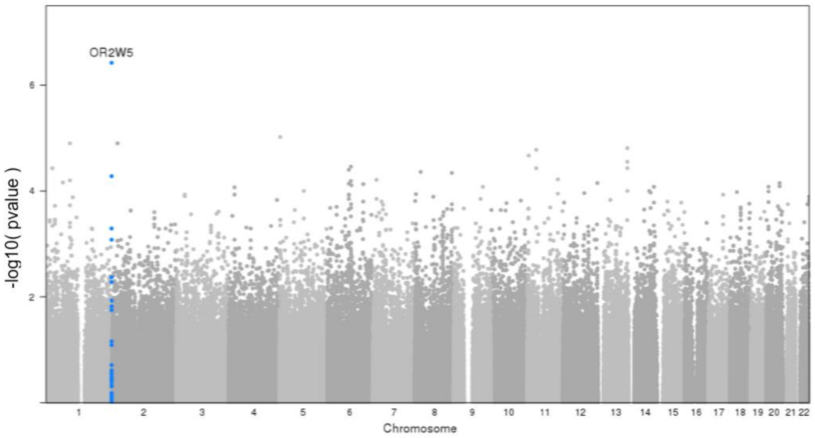


**Supplementary Fig. 8. Manhattan plot of gustin GWAS during LCD intervention.** The Manhattan plot displays GWA results for change in gustin protein level during LCD. SNPs located in the regulatory region of *OR2W5* are highlighted in blue. Best-associated SNP rs7512601 was associated with protein level change during LCD with nominal p = 3.8 x 10-7.

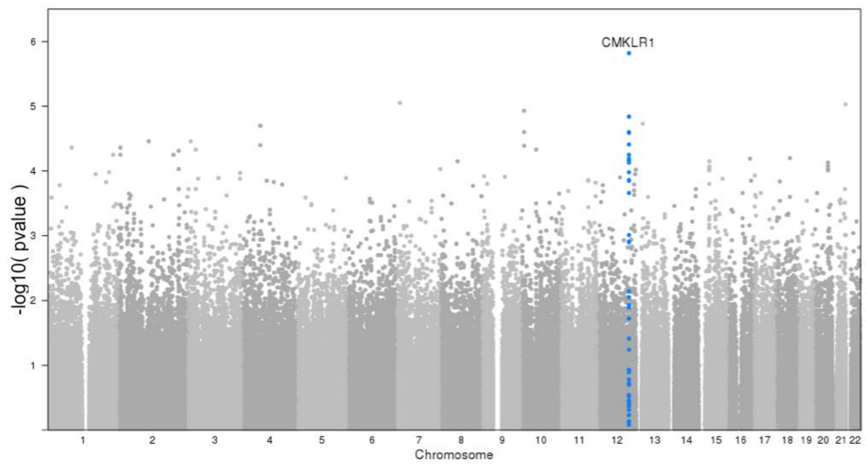


**Supplementary Fig. 9. Manhattan plot of chemerin GWAS during LCD intervention.** The Manhattan plot displays GAW results for change in chemerin protein level during LCD. SNPs located in the regulatory region of *CMKLR1* are highlighted. Best-associated SNP rs11113832 was associated with protein level change during LCD with nominal p = 1.5 x $10^{-6}$.

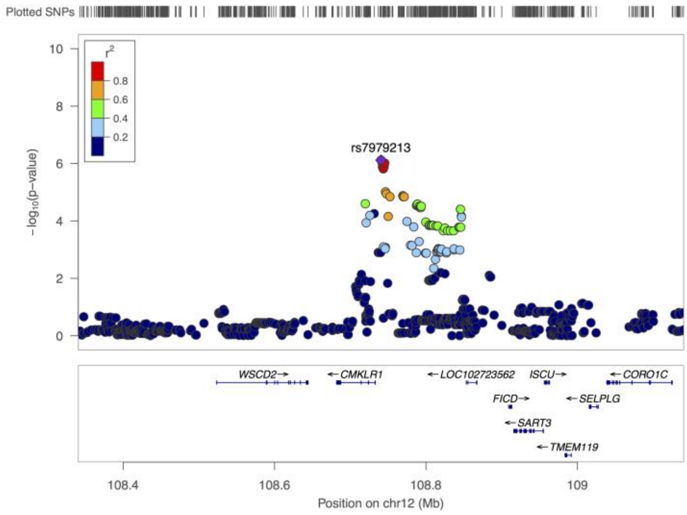


**Supplementary Fig. 10. Locus-specific plots for chemerin *trans*-acting SNPs within *CMKLR1* region during LCD intervention.** Strongest association to protein level change during LCD estimated for SNP rs11113832 with $p = 1.5 \times 10^{-6}$ located in the promoter region of olfactory receptor gene *CMKLR1.*

a

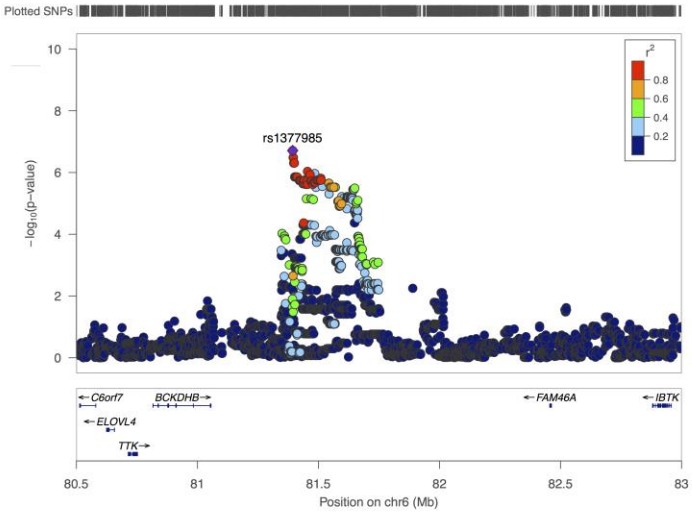


b

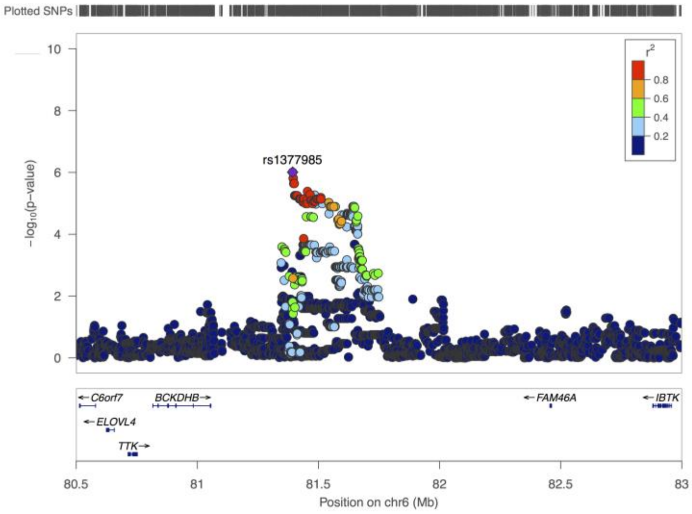


**Supplementary Fig. 11. Locus-specific plots for *trans*-acting SNPs of HSP70 protein expression variation during LCD located in between *BCKDHB* and *FAM46A* genes**. Association plot for *trans*-pQTL SNPs associated with (a) HSP70 protein expression change; then (b) adding leptin protein expression variation during LCD as a confounder in the linear mixed model.

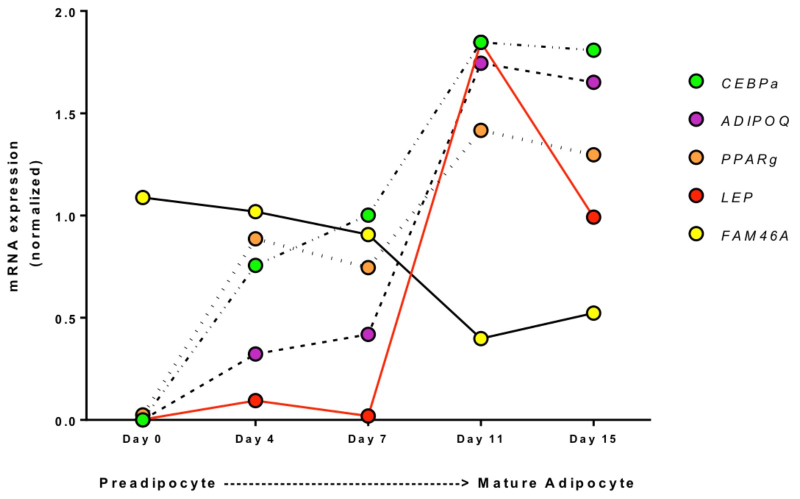


**Supplementary Fig. 12. *FAM46A*, *LEP*, *PPAR*γ, *CEBP*α and *ADIPOQ* expression during adipocyte differentiation**. Relative mRNA expression for FAM46A and LEP were measured every four days from day 0 of differentiation to full maturation at day 15. The mRNA expression was determined by quantitative real-time PCR (qRT-PCR).

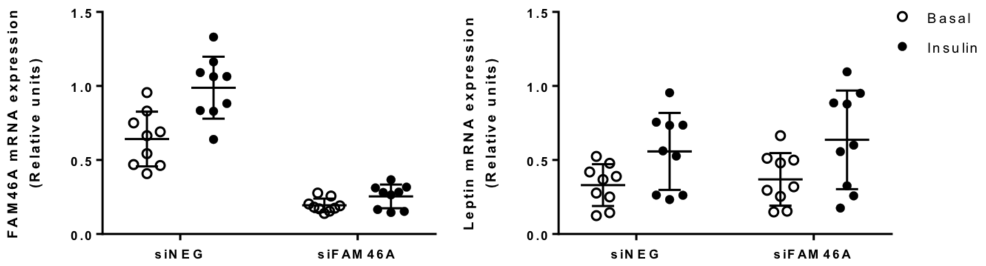


**Supplementary Fig. 13.** ***FAM46A*** **gene silencing effect on leptin protein expression in SGBS adipocytes.** Individual data points corresponding to fig. 4A and 4B in the main manuscript. SGBS cells were transfected with negative control siRNA (siNEG) or *FAM46A*-specific siRNA (siFAM46A) and experiments were performed at day nine. Gene expression levels are expressed relative to TBP (TATA Binding Protein) expression. Results are given as the mean +/-SD for n=9.

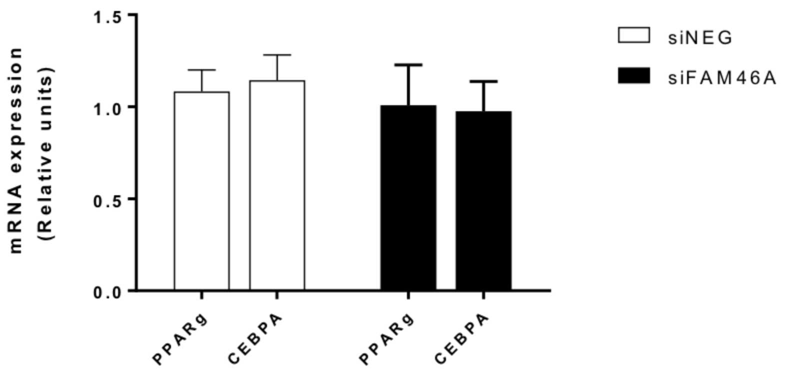


**Supplementary Fig. 14. Expression levels of *PPAR*γ and *CEBP*α in the wildtype and knockdown SGBS adipocytes.** SGBS cells transfected with negative control siRNA (siNEG) or *FAM46A*-specific siRNA (siFAM46A). *PPAR*γ and CEBPα mRNA was assessed by quantitative real-time PCR (qRT-PCR) at day nine. Gene expression levels are expressed relative to TBP (TATA Binding Protein) gene expression. The data are shown as the means +/- SD (n=5). *FAM46A* knockdown did not affect either *PPAR*γ or *CEBP*α expression levels (Student's *t* test $p_{ppar\gamma}$= 0.4789, $p_{CEBP\alpha}$= 0.0821, n=6).

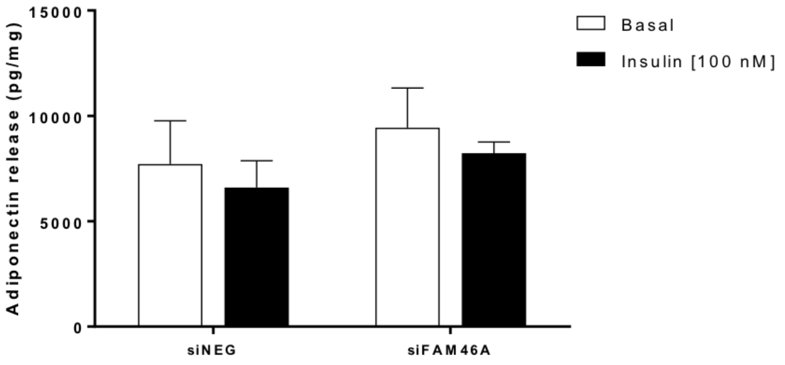


**Supplementary Fig. 15. Secretion of adiponectin in the wildtype and knockdown SGBS adipocytes.** SGBS cells were transfected with negative control siRNA (siNEG) or *FAM46A*-specific siRNA (siFAM46A). Adiponectin secretion was determined by enzyme-linked immuneabsorbant assay (ELISA) at baseline or after stimulation with 100 nM insulin for 6 hours. The data are shown as the means +/- SD (n=3) for statistical significance calculated using the Student's *t* test. Adiponectin secretion did not differ between wildtype and *FAM46A* knockdown (Student's *t* test $p_{Basal} = 0.3453$, $p_{Insulin} = 0.1095$, n=3).

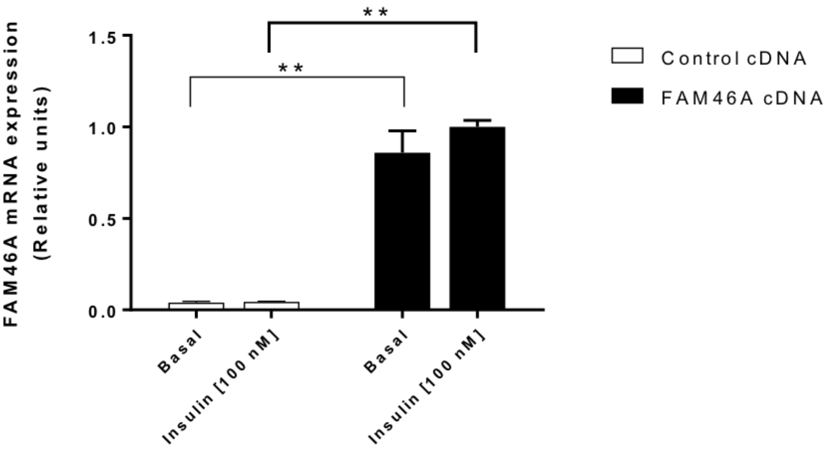


**Supplementary Fig. 16. Overexpression of *FAM46A* in SGBS adipocytes.** SGBS cells were transfected with 0.5 µg full length *FAM46A* cDNA for 48h and resulted in a 20-fold increase of *FAM46A* expression. *FAM46A* mRNA was assessed by qRT/PCR at day nine. Gene expression levels are expressed relative to *TBP* (TATA Binding Protein) expression. The data are shown as the means +/- SD (n=3) with *P<0.05, **P<0.01 for statistical significance calculated using the Student's *t* test ($p_{Basal}$ = 3.0 x 10-4, $p_{Insulin}$= 1.0 x 10-4).

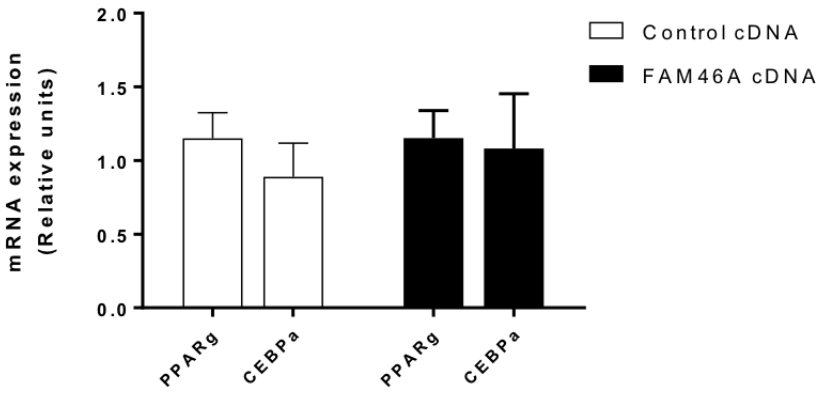


**Supplementary Fig. 17. Expression levels of *PPAR*γ and *CEBP*α in the wildtype and *FAM46A* overexpressing SGBS adipocytes.** mRNA levels of *PPAR*γ and *CEBP*α were measured by qRT-PCR. Gene expression levels are expressed relative to *TBP* (TATA Binding Protein) expression. *PPAR*γ and *CEBP*α levels did not differ between wildtype and *FAM46A* overexpressing cells ($p_{PPARg}$ = 0.9813, $p_{CEBP\alpha}$ = 0.3939, n=5). Data are shown as the means +/- SD.

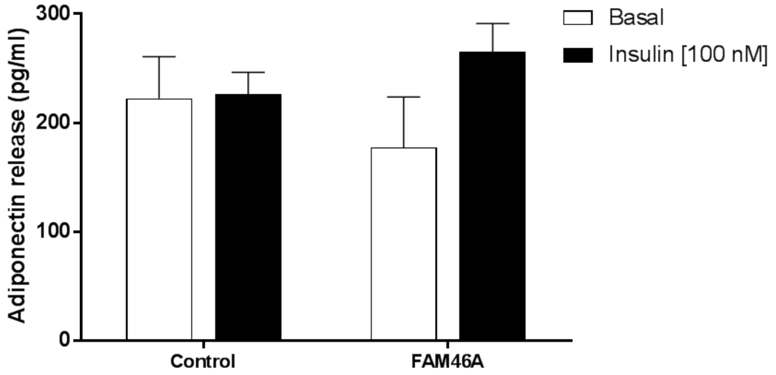


**Supplementary Fig. 18. Secretion of adiponectin in the wildtype and FAM46A overexpressing SGBS adipocytes.** SGBS cells were transfected with 0.5 µg of empty vector (Control) or FAM46A-specific cDNA (*FAM46A*). Adiponectin and IL6 secretion were determined by enzyme-linked immunoabsorbant assay (ELISA) at baseline or after stimulation with 100 nM insulin for 6 hours. Adiponectin secretion did not differ between wildtype and *FAM46A* knockdown (Student's *t* test $p_{Basal} = 0.26$, $p_{Insulin} = 0.11$, n=3). The data are shown as the means +/- SD.

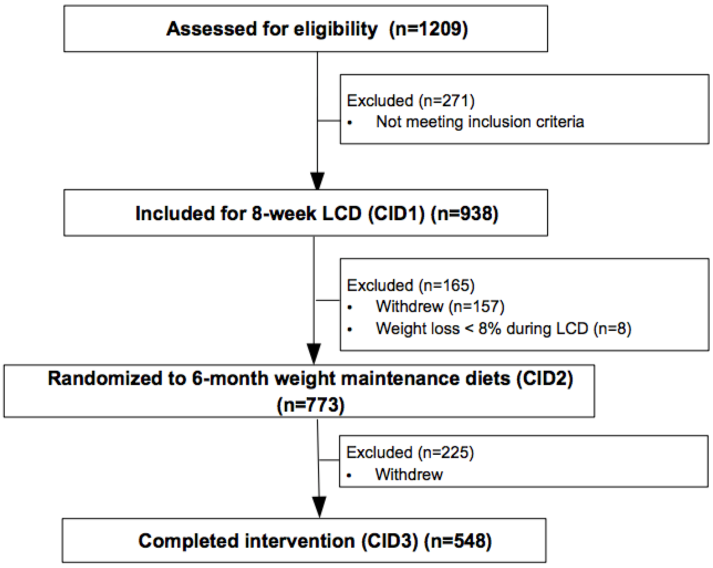


**Supplementary Fig. 19. Flowchart for the selection of participants to the Diogenes intervention study.**

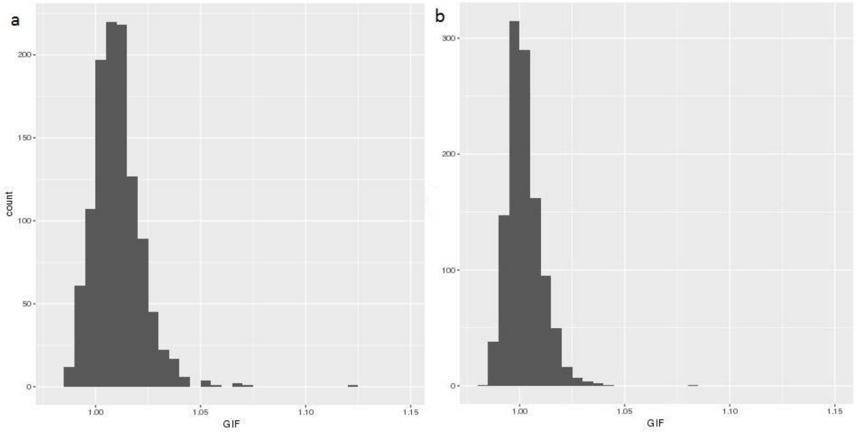


**Supplementary Fig. 20. pQTL genomic inflation factors histograms.** Distribution of genomic inflation factors estimated from 1,129 pQTL studies using protein expressions at (a) baseline and (b) during LCD intervention**.**

**Supplementary Table 1. BMI association results from Sun et al.[21] for 38 proteins associated with BMI at baseline and during LCD in Diogenes.** Association results available in Supplementary Table 17 from Sun et al.[21] testing association between BMI and Somalogic protein levels in a population-based cohort for 38 proteins associated with BMI at baseline and during LCD in current Diogenes study. P-values were corrected for multiple testing using the Benjamini-Hochberg method in the Diogenes proteome-wide study. Assuming a Bonferroni corrected nominal P-value of 0.0012 (0.05 / 42 tested proteins for replication in Sun et al.) all proteins replicated their association to BMI in Sun et al.[21]. Coeff., estimated association coefficient; SE, standard error; P-value, association P-value; Corrected P-value, association P-value corrected for multiple testing.

| | | Diogenes Proteome-Wide | | | | Sun et al. | | |
|---|---|---|---|---|---|---|---|---|
| **Protein** | **Coding gene** | **Coeff.** | **SE** | **P-value** | **Corrected P-value** | **Coeff.** | **SE** | **P-value** |
| **Leptin** | **LEP** | 4.76 | 0.33 | 3.22E-40 | 3.64E-37 | 0.118 | 0.002 | 3.98E-402 |
| **WFKN2** | **WFIKKN2** | -3.07 | 0.55 | 4.97E-08 | 1.81E-06 | -0.088 | 0.003 | 7.94E-133 |
| **IGFBP-2** | **IGFBP2** | -2.07 | 0.43 | 2.00E-06 | 4.70E-05 | -0.086 | 0.003 | 5.01E-132 |
| **Growth hormone receptor** | **GHR** | 2.60 | 0.55 | 3.47E-06 | 7.54E-05 | 0.083 | 0.003 | 3.98E-121 |
| **IGFBP-1** | **IGFBP1** | -2.07 | 0.23 | 1.17E-17 | 2.64E-15 | -0.084 | 0.003 | 6.31E-120 |
| **SAP** | **APCS** | 4.18 | 0.89 | 3.83E-06 | 8.17E-05 | 0.074 | 0.003 | 3.98E-104 |
| **Nogo Receptor** | **RTN4R** | 2.72 | 0.71 | 0.00013 | 0.00174 | 0.079 | 0.004 | 1.26E-103 |
| **UNC5H4** | **UNC5D** | -4.74 | 0.55 | 1.18E-16 | 1.67E-14 | -0.063 | 0.004 | 6.31E-68 |
| **Endocan** | **ESM1** | -2.34 | 0.59 | 7.60E-05 | 0.00110 | -0.058 | 0.004 | 3.16E-54 |
| **IL-1 R AcP** | **IL1RAP** | -1.75 | 0.48 | 0.00034 | 0.00376 | -0.057 | 0.004 | 2.51E-51 |
| **CRDL1** | **CHRDL1** | -2.88 | 0.77 | 0.00021 | 0.00258 | -0.053 | 0.004 | 1.58E-46 |
| **Antithrombin III** | **SERPINC1** | -11.95 | 1.36 | 3.13E-17 | 5.88E-15 | -0.052 | 0.004 | 1.26E-45 |
| **TSG-6** | **TNFAIP6** | -1.75 | 0.48 | 0.00027 | 0.00312 | -0.051 | 0.004 | 2.51E-45 |
| **SCF sR** | **KIT** | -2.24 | 0.55 | 5.35E-05 | 0.00085 | -0.05 | 0.004 | 1.58E-44 |
| **SHBG** | **SHBG** | -1.68 | 0.29 | 7.49E-09 | 3.38E-07 | -0.088 | 0.006 | 3.16E-43 |
| **C1s** | **C1S** | 10.06 | 1.10 | 2.05E-18 | 5.80E-16 | 0.096 | 0.007 | 2.51E-42 |
| **TFPI** | **TFPI** | 2.64 | 0.68 | 0.00013 | 0.00174 | 0.042 | 0.003 | 3.98E-37 |
| **WIF-1** | **WIF1** | -3.02 | 0.74 | 5.66E-05 | 0.00089 | -0.047 | 0.004 | 7.94E-37 |
| **tPA** | **PLAT** | 1.93 | 0.52 | 0.00022 | 0.00268 | 0.053 | 0.004 | 3.98E-36 |
| **Insulin** | **INS** | 1.33 | 0.39 | 0.00080 | 0.00755 | 0.04 | 0.004 | 1.26E-25 |

| | | | | | | | | |
|---|---|---|---|---|---|---|---|---|
| **Carbonic anhydrase 6** | **CA6** | -0.86 | 0.28 | 0.00220 | 0.01765 | -0.036 | 0.004 | 2.00E-24 |
| **sE-Selectin** | **SELE** | 1.11 | 0.41 | 0.00682 | 0.04153 | 0.038 | 0.004 | 5.01E-24 |
| **TECK** | **CCL25** | 0.93 | 0.29 | 0.00141 | 0.01195 | 0.038 | 0.004 | 1.26E-23 |
| **Coagulation Factor IX** | **F9** | 6.65 | 1.10 | 3.12E-09 | 1.76E-07 | 0.07 | 0.007 | 3.98E-21 |
| **Coagulation Factor IXab** | **F9** | 6.65 | 1.10 | 3.12E-09 | 1.76E-07 | 0.069 | 0.007 | 7.94E-21 |
| **Haptoglobin** | **Mixed Type** | 1.09 | 0.27 | 6.95E-05 | 0.00105 | 0.035 | 0.004 | 6.31E-20 |
| **ATS13** | **ADAMTS13** | -2.19 | 0.63 | 0.00056 | 0.00556 | -0.032 | 0.004 | 2.00E-18 |
| **bFGF-R** | **FGFR1** | -3.64 | 0.91 | 6.74E-05 | 0.00103 | -0.058 | 0.007 | 3.16E-15 |
| **PAI-1** | **SERPINE1** | 1.50 | 0.26 | 1.38E-08 | 5.76E-07 | 0.029 | 0.004 | 3.16E-14 |
| **C7** | **C7** | -3.01 | 0.77 | 0.00011 | 0.00153 | -0.051 | 0.007 | 5.01E-12 |
| **RET** | **RET** | 1.58 | 0.35 | 8.40E-06 | 0.00016 | 0.022 | 0.003 | 1.58E-11 |
| **CATZ** | **CTSZ** | 2.89 | 0.65 | 1.10E-05 | 0.00021 | 0.025 | 0.004 | 2.00E-11 |
| **BCMA** | **TNFRSF17** | -2.07 | 0.70 | 0.00349 | 0.02464 | -0.026 | 0.004 | 2.00E-11 |
| **GDF-11** | **GDF11** | -2.76 | 0.74 | 0.00024 | 0.00289 | 0.024 | 0.004 | 3.16E-11 |
| **LG3BP** | **LGALS3BP** | 1.60 | 0.45 | 0.00045 | 0.00480 | 0.038 | 0.007 | 1.26E-07 |
| **HPT** | **HP** | 1.09 | 0.27 | 6.95E-05 | 0.00105 | 0.038 | 0.007 | 2.00E-07 |
| **IL-17B R** | **IL17RB** | -0.78 | 0.29 | 0.00765 | 0.04568 | -0.019 | 0.004 | 3.16E-07 |
| **Angiopoietin-2** | **ANGPT2** | 3.77 | 0.58 | 1.65E-10 | 1.43E-08 | -0.018 | 0.004 | 2.00E-06 |

**Supplementary Table 2. Population description.** Description of 494 subjects included in pQTL and 151 in eQTL association studies.

| Study | Gender | Age range | Centers | BMI baseline range | BMI fold change range |
|---|---|---|---|---|---|
| **pQTL (n = 494)** | 316 females / 178 males | 16 – 63 | Denmark = 98<br>Netherland = 86<br>Czech Republic = 69<br>Spain = 67<br>Bulgaria = 55<br>Greece = 45<br>Germany = 35<br>UK = 39 | 25.6 – 52.0 | 0.78 - 0.95 |
| **eQTL (n = 151)** | 98 females / 53 males | 24 - 63 | Denmark = 28<br>Netherland = 35<br>Czech Republic = 1<br>Spain = 40<br>Bulgaria = 12<br>Greece = 8<br>Germany = 17<br>UK = 10 | 27.1 – 47.7 | 0.78 – 0.94 |

## Supplementary Table 3

**Primer sequences.** Forward and reverse primer sequences.

| GENE NAME | PRIMER SEQUENCE |
|---|---|
| ***TBP (*HOUSEKEEPING GENE)** | Forward primer - TGGTGTGCACAGGAGCCAAG<br>Reverse primer - TTCACATCACAGCTCCCCAC |
| ***LEP*** | Forward primer - GGCTTTGGCCCTATCTTTTC<br>Reverse primer - ACCGGTGACTTTCTGTTTGG |
| ***FAM46A*** | Forward primer - CAACAGTGGCAAAAATGTGG<br>Reverse primer - TCCTGGAAATCGCCATAGAC |
| ***PPARΓ*** | Forward primer - GATCCAGTGGTTGCAGATTACAA<br>Reverse primer - GAGGGAGTTGGAAGGCTCTTC |
| ***CEBP$\alpha$*** | Forward primer - TGGACAAGAACAGCAACGAG<br>Reverse primer - TTGTCACTGGTCAGCTCCAG |
| ***ADIPOQ*** | Forward primer - GGCCGTGATGGCAGAGAT<br>Reverse primer - CCTTCAGCCCGGGTACT |

**Supplementary References**


1. Corominola, H. et al. Identification of novel genes differentially expressed in omental fat of obese subjects and obese type 2 diabetic patients. Diabetes 50, 2822–2830 (2001).

2. Gabrielsson, B. G. et al. High Expression of Complement Components in Omental Adipose Tissue in Obese Men. Obes. Res. 11, 699–708 (2003).

3. Chiellini, C. et al. Obesity modulates the expression of haptoglobin in the white adipose tissue via TNFalpha. J. Cell. Physiol. 190, 251–258 (2002).

4. Chiellini, C. et al. Serum Haptoglobin: A Novel Marker of Adiposity in Humans. J. Clin. Endocrinol. Metab. 89, 2678–2683 (2004).

5. Glowinska, B., Urban, M., Peczynska, J. & Florys, B. Soluble adhesion molecules (sICAM-1, sVCAM-1) and selectins (sE selectin, sP selectin, sL selectin) levels in children and adolescents with obesity, hypertension, and diabetes. Metabolism. 54, 1020–1026 (2005).

6. Ito, H. et al. Weight reduction decreases soluble cellular adhesion molecules in obese women. Clin. Exp. Pharmacol. Physiol. 29, 399–404 (2002).

7. Roberts, C. K., Chen, A. K. & Barnard, R. J. Effect of a short-term diet and exercise intervention in youth on atherosclerotic risk factors. Atherosclerosis 191, 98–106 (2007).

8. Hanzu, F. A. et al. Translational evidence of endothelial damage in obese individuals: inflammatory and prothrombotic responses. J. Thromb. Haemost. JTH 9, 1236–1245 (2011).

9. Bozaoglu, K. et al. Plasma Levels of Soluble Interleukin 1 Receptor Accessory Protein Are Reduced in Obesity. J. Clin. Endocrinol. Metab. 99, 3435–3443 (2014).

10. Guo, X. et al. [Expression of TSG-6 gene during 3T3-L1 preadipocyte differentiation and regulative role of tumor necrosis factor-alpha]. Zhonghua Er Ke Za Zhi Chin. J. Pediatr. 42, 344–347 (2004).

11. Goralski, K. B. et al. Chemerin, a Novel Adipokine That Regulates Adipogenesis and Adipocyte Metabolism. J. Biol. Chem. 282, 28175–28188 (2007).

12. Li, Y., Shi, B. & Li, S. Association between Serum Chemerin Concentrations and Clinical Indices in Obesity or Metabolic Syndrome: A Meta-Analysis. PLOS ONE 9, e113915 (2014).

13. Crawley, J. T. B., Lane, D. A., Woodward, M., Rumley, A. & Lowe, G. D. O. Evidence that high von Willebrand factor and low ADAMTS-13 levels independently increase the risk of a non-fatal heart attack. J. Thromb. Haemost. 6, 583–588 (2008).

14. Liu, M. Y. et al. Gender-dependent up-regulation of ADAMTS-13 in mice with obesity and hypercholesterolemia. Thromb. Res. 129, 536–539 (2012).

15. Shitaye, H. S., Terkhorn, S. P., Combs, J. A. & Hankenson, K. D. Thrombospodin-2 is an endogenous adipocyte inhibitor. Matrix Biol. J. Int. Soc. Matrix Biol. 29, 549–556 (2010).

16. Voros, G. et al. Modulation of Angiogenesis during Adipose Tissue Development in Murine Models of Obesity. Endocrinology 146, 4545–4554 (2005).

17. Nadler, S. T. et al. The expression of adipogenic genes is decreased in obesity and diabetes mellitus. Proc. Natl. Acad. Sci. U. S. A. 97, 11371–11376 (2000).

18. Padiglia, A. et al. Sensitivity to 6-n-propylthiouracil is associated with gustin (carbonic anhydrase VI) gene polymorphism, salivary zinc, and body mass index in humans. Am. J. Clin. Nutr. 92, 539–545 (2010).

19. Kondás, K., Szláma, G., Trexler, M. & Patthy, L. Both WFIKKN1 and WFIKKN2 Have High Affinity for Growth and Differentiation Factors 8 and 11. J. Biol. Chem. 283, 23677–23684 (2008).

20. Allen, D. L., Hittel, D. S. & McPherron, A. C. Expression and Function of Myostatin in Obesity, Diabetes, and Exercise Adaptation. Med. Sci. Sports Exerc. 43, 1828–1835 (2011).

21. Sun, Benjamin B., et al. "Consequences Of Natural Perturbations In The Human Plasma Proteome." bioRxiv, 134551 (2017).

22. Suhre, Karsten, et al. Connecting genetic risk to disease end points through the human blood plasma proteome. Nature communications 8, 14357 (2017).

23. Locke, A. E. et al. Genetic studies of body mass index yield new insights for obesity biology. Nature 518, 197–206 (2015).
