# Supplementary material for: Protein quantitative trait locus study in obesity during weight-loss identifies a leptin regulator
Source: Nat Commun. 2017 Dec 12;8:2084. doi: 10.1038/s41467-017-02182-z (PMC5727191; doi:10.1038/s41467-017-02182-z)
Supplement: Supplementary file 3 — Description of Additional Supplementary Files [file 41467_2017_2182_MOESM3_ESM.docx]

**Description of Supplementary Data files**

**Supplementary Data 1.**

**Statistical test results for the association of protein expression with BMI at baseline**. Results from association test (linear regression adjusted on age, gender and center) between expression of 1,129 proteins and BMI at baseline with information about name of the coding gene. Coefficient, estimated association coefficient; SE, standard error; Pvalue, association P-value; Corrected Pvalue, association P-value corrected for multiple testing using Benjamini-Hochberg method.

**Supplementary Data 2.**

**Statistical test results for the association of protein expression change during LCD with BMI.** Results from association test (linear regression adjusted on age, gender and center) between expression of 1,129 proteins and BMI during LCD intervention with information about name of the coding genes. Coefficient, estimated association coefficient; SE, standard error; Pvalue, association P-value; Corrected Pvalue, association P-value corrected for multiple testing using Benjamini-Hochberg method.

**Supplementary Data 3.**

**Replication of Diogenes pQTL results in two population based studies available from Sun et al. (2017) and Suhre et al (2017).** Ten Diogenes pQTL identified at baseline replicated their association signal in two population-based cohorts; 8 in the KORA cohort in Suhre et al.^22^ and 2 in a subset of the INTERVAL study cohort available in Sun et al^21^. For each SNP, chromosome and position in bp is provided with minor allele and corresponding frequency, association coefficient, corresponding P-values and regulation as published in the two population-based studies^21,22^ or reported in the current Diogenes study. Replication in the QMDiab replication cohort^21^ is also reported. A1, tested allele; Freq, tested allele frequency; Coeff, estimated association coefficient; P, association P-value.

* Replication in QMDiab cohort of pQTL signal identified in KORA cohort

** pQTL association results based on Diogenes baseline data

**Supplementary Data 4.**

**Summary statistics for 22 Diogenes pQTL replicated in two population-based cohort available from Suhre et al. (2017) and Sun et al (2017).** pQTL association results for 22 Diogenes pQTL SNPs or SNPs in LD (r2 > 0.2) available from 2 population-based pQTL association studies^21,22^. Minor allele, corresponding frequency, estimated coefficient and P-value from association test and regulation are reported for Diogenes pQTL. Association results for corresponding SNPs or SNPs in LD with r2 > 0.2 with position (chromosome and position in bp) together with minor allele, frequency and regulation are provided as reported in Sun et al.^21^ and Suhre et al^22^. A1, tested allele; Freq, tested allele frequency; Coeff, estimated association coefficient; P, association P-value; r2, linkage disequilibrium coefficient.

* Diogenes pQTL SNP association results with pQTL SNP in LD in Suhre et al.^22^ pQTL study

** Diogenes pQTL SNP association results with pQTL SNP in LD in Sun et al.^21^ pQTL study

§ SNP in linkage disequilibrium (LD; r2 > 0.2) with Diogenes pQTL and identified as a pQTL for the same protein in Suhre et al^22^.

**Supplementary Data 5.**

**Proteins description.** Description of 14 proteins associated with BMI at baseline and during LCD genetically regulated at baseline. Official full name and summary information about protein coding genes was available from the National Center for Biotechnology Information's (NCBI) Gene database.

**Supplementary Data 6.**

**Comparison of baseline pQTL with Locke et al. (2015) GWAS results.** Association results from 16 pQTL identified at baseline nominally associated with BMI in Locke et al.^23^ GWAS. Freq, minor allele frequency; Coef, estimated association coefficient; P, association P-value.

*Different minor alleles were observed between the two studies for 2 SNPs in linkage disequilibrium (rs3802967 and rs4150581). Using 1000 Genomes Project Phase 3 allele frequencies (via <http://www.ensembl.org/>), C and G allele frequencies were estimated to 0.54 for rs3802967 and rs4150581, close to 0.50 frequencies observed in our study.

**Supplementary Data 7.**

**Baseline pQTL association results from BMI-associated SNPs identified in Locke et al (2015) GWAS.** Information from 62 SNPs was available. Missing SNPs were replaced by SNPs in LD (r2 > 0.8) for 21 SNPs leading to 83 SNPs with pQTL results. For each protein, pQTL association results for this set of SNPs were extracted and P-values corrected for multiple testing using Benjamin-Hochberg method. For each SNP, the stronger pQTL signal is provided. For each SNP, the position (in bp) and the chromosome, with the minor allele and corresponding frequency is provided. If not genotyped in the current pQTL study, a proxy SNP (if available) with pairwise linkage disequilibrium (r2) is provided. The name of protein with the strongest association signal for the SNP (or its proxy) with corresponding coding gene and association results are available. Information about cis / trans-regulation of the QTL is also provided with the name of the nearest gene. Freq, allele frequency; Coef, estimated association coefficient; P, association P-value; Corrected P, association P-value corrected for multiple testing using Benjamini-Hochberg method.

**Supplementary Data 8.**

**LCD pQTL association results from BMI-associated SNPs identified in Locke et al. (2015) GWAS.** Information from 62 SNPs was available. Missing SNPs were replaced by SNPs in LD (r2 > 0.8) for 21 SNPs leading to 83 SNPs with pQTL results. For each protein, pQTL association results for this set of SNPs were extracted and P-values corrected for multiple testing using Benjamin-Hochberg method. For each SNP, the stronger pQTL signal is provided. For each SNP, the position (in bp) and the chromosome, with the minor allele and corresponding frequency is provided. If not genotyped in the current pQTL study, a proxy SNP (if available) with pairwise linkage disequilibrium (r2) is provided. The name of protein with the strongest association signal for the SNP (or its proxy) with corresponding coding gene and statistical association results are available. Information about cis / trans-regulation of the QTL is also provided with the name of the nearest gene. Freq, allele frequency; Coef, estimated association coefficient; P, association P-value; Corrected P, association P-value corrected for multiple testing using Benjamini-Hochberg method.
